# Structural Basis of Pan-Ebolavirus Neutralization by a Human Antibody against a Conserved, yet Cryptic Epitope

**DOI:** 10.1128/mBio.01674-18

**Published:** 2018-09-11

**Authors:** Brandyn R. West, Crystal L. Moyer, Liam B. King, Marnie L. Fusco, Jacob C. Milligan, Sean Hui, Erica Ollmann Saphire

**Affiliations:** a Department of Immunology and Microbiology, Scripps Research, La Jolla, California, USA; b Skaggs Institute for Chemical Biology, Scripps Research, La Jolla, California, USA; Icahn School of Medicine at Mount Sinai

**Keywords:** Bundibugyo virus, Ebola virus, antibody, broadly neutralizing, glycoprotein, pan-ebolavirus, structure

## Abstract

Only one naturally occurring human antibody has been described thus far that is capable of potently neutralizing all five ebolaviruses. Here we present two crystal structures of this rare, pan-ebolavirus neutralizing human antibody in complex with Ebola virus and Bundibugyo virus glycoproteins (GPs), respectively. The structures delineate the key protein and glycan contacts for binding that are conserved across the ebolaviruses, explain the antibody’s unique broad specificity and neutralization activity, and reveal the likely mechanism behind a known escape mutation in the fusion loop region of GP2. We found that the epitope of this antibody, ADI-15878, extends along the hydrophobic paddle of the fusion loop and then dips down into a highly conserved pocket beneath the N-terminal tail of GP2, a mode of recognition unlike any other antibody elicited against Ebola virus, and likely critical for its broad activity. The fold of Bundibugyo virus glycoprotein, not previously visualized, is similar to the fold of Ebola virus GP, and ADI-15878 binds to each virus’s GP with a similar strategy and angle of attack. These findings will be useful in deployment of this antibody as a broad-spectrum therapeutic and in the design of immunogens that elicit the desired broadly neutralizing immune response against all members of the ebolavirus genus and filovirus family.

## Abstract (precis)

There are five different members of the *Ebolavirus* genus. Provision of vaccines and treatments able to protect against any of the five ebolaviruses is an important goal of public health. Antibodies are a desired result of vaccines and can be delivered directly as therapeutics. Most antibodies, however, are effective against only one or two, not all, of these pathogens. Only one human antibody has been thus far described to neutralize all five ebolaviruses, antibody ADI-15878. Here we describe the molecular structure of ADI-15878 bound to the relevant target proteins of Ebola virus and Bundibugyo virus. We explain how it achieves its rare breadth of activity and propose strategies to design improved vaccines capable of eliciting more antibodies like ADI-15878.

## INTRODUCTION

The unexpected location and unprecedented scale of the 2013–2016 Ebola virus pandemic underscored the need for provision of therapeutics and vaccines. A major practical challenge, however, is that there are five antigenically distinct viruses in the *Ebolavirus* genus. Four of the five viruses are known to cause severe disease in humans. The first, Ebola virus (EBOV), was linked in 2014 to 2016 to a 28,000-person outbreak with 41% lethality (1) and emerged again once in 2017 and twice in 2018 in the Democratic Republic of the Congo. The second, Bundibugyo virus (BDBV), emerged in 2007 (2, 3) and again in 2012 (2, 3) with ∼25 to 50% lethality. The third, Sudan virus (SUDV), has emerged at least six times among humans (4–6), typically with 50% lethality. The fourth, Taï Forest ebolavirus (TAFV), emerged in a veterinarian working in West Africa in 1994 (7); it is unknown whether or where it might emerge again. The fifth, Reston virus (RESTV), has not caused disease in the few humans exposed, but it is highly lethal to nonhuman primates (8) and has emerged in multiple ranches of swine raised for human food in China and the Philippines (9–11). Viruses of the *Marburgvirus* genus, also members of the *Filovirus* family, cause human diseases resembling those of the ebolaviruses (12, 13).

Antibody therapeutics are an attractive emergency postexposure treatment or preexposure prophylaxis strategy for these viruses. An obstacle, however, is that the five different ebolaviruses are antigenically distinct. The surface glycoprotein GP is up to 59% different in amino acid sequence across the ebolaviruses, although some functional domains of GP exhibit a higher degree of conservation. To date, only one naturally occurring human antibody has been described that is able to neutralize all of them. This antibody, termed ADI-15878, was identified in a human survivor of the 2013–2016 outbreak (14). ADI-15878 is escaped by point mutation G528E located on the paddle of the Ebola virus GP fusion loop (15). A murine antibody, termed 6D6 (15, 16), and a macaque antibody, termed CA-45 (17), are also thought to bind the fusion loop and achieve pan-ebolavirus reactivity by doing so. No structure is yet available for Bundibugyo virus GP or any human pan-ebolavirus antibody. Therefore, it is unclear how broad recognition and activity are achieved. It is also unclear precisely how recognition by such a pan-ebolavirus antibody differs from recognition by competing, monospecific antibodies that recognize overlapping epitopes, but which recognize only Ebola virus or only Sudan virus.

All five ebolaviruses express a single glycoprotein on their surface, termed GP. In producer cells, the GP precursor is cleaved by furin to yield two subunits: GP1, which mediates receptor binding, and GP2, which mediates fusion (18–24). GP1 and GP2 are linked by a disulfide bond, and the GP1-GP2 pair assembles into a trimer of GP1-GP2 heterodimers on the viral surface (25, 26). In the GP2 fusion subunit is an N-terminal tail that docks along the side of GP after furin cleavage, an internal fusion loop, and two heptad repeats. The fusion loop forms an antiparallel beta strand pair with a hydrophobic loop between the two beta strands (this forms a structure that looks like a “paddle” in ribbon representations of GP) which packs into the neighboring monomer in the assembled trimer (25–27). During viral entry, the fusion loop must unwrap from its position in the assembled trimer to project into the target cell’s membrane. Nuclear magnetic resonance (NMR) structures of the fusion loop suggest that during entry, upon exposure to endosomal pH, the central loop changes conformation from an open paddle to condense into a fist-like structure (18, 19). During the entry process, GP is processed in the endosome by cathepsin enzymes (28–30) to delete a mucin-like domain and glycan cap domain from the GP1 subunit (31, 32). The cleaved GP, termed GP_CL_, is then potentiated for receptor binding and viral/endosomal membrane fusion (22, 33–35). The five ebolavirus GPs are ∼41% identical overall and ∼68% identical in the GP_CL_ core outside the glycan cap and mucin-like domain. Of the five ebolaviruses, structures are thus far available only for Ebola virus (22, 25, 26, 35–37) and Sudan virus GPs (27, 38).

Here we describe crystal structures of ADI-15878 in complex with cleaved Ebola virus and cleaved Bundibugyo virus GPs. The crystal structures reveal that ADI-15878 binds to the base of the glycoprotein spike. However, unlike other antibodies elicited against Ebola virus, ADI-15878 achieves broad specificity by binding underneath the N-terminal tail of the GP2 fusion subunit and into a cryptic hydrophobic pocket underneath (N-terminal [N-term] pocket). The majority of its contacts are to conserved residues of GP in and around this pocket, as well as a glycan linked to GP residue N563 nearby. The NXT sequon and presence of this glycan are completely conserved across the *Ebolavirus* genus. The variable heavy (V_H_) chain of ADI-15878 is only ∼6% different in primary sequence from the V_H_ segment of its germ line precursor (VH3-23) (15). The similarity to germ line and the moderate length of its complementarity-determining region (CDR) H3 (15 amino acids) suggest that ADI-15878 could be a prototype of other broadly neutralizing antibodies which could be elicited by vaccination.

## RESULTS

**FIG 1:**
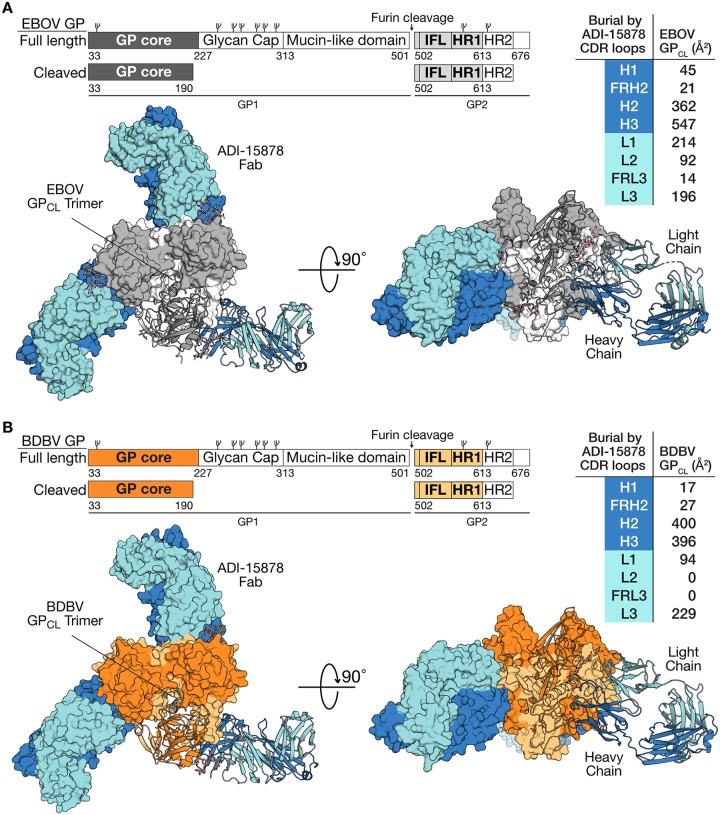
GP organization and ADI-15878 complexes. (A) Schematic comparing full-length GP and cleaved EBOV GP (GP_CL_), and crystal structure of the ADI-15878–EBOV GP_CL_ complex. Components are indicated by different colors. GP_CL_ is gray. The ADI-15878 light chain is light blue, and the heavy chain is dark blue. Top and side views are shown. One monomer is shown in a ribbon format, and the other two are shown as molecular surfaces. The buried surface contributed by each CDR is indicated in the table to the right. IFL, internal fusion loop; HR1, heptad repeat 1. (B) Schematic of BDBV GP and GP_CL_ and the ADI-15878–BDBV GP_CL_ crystal structure. BDBV GP_CL_ is shown in orange, with GP1 dark orange and GP2 light orange. The ADI-15878 light chain is light blue, and the heavy chain is dark blue. The values for CDR buried surface area in the table differ significantly between EBOV and BDBV especially within CDRs H3 and L2 and framework region L3. This difference is due to the lack of density for the GP2 glycan at position N563 in the lower-resolution ADI-15878–BDBV GP complex structure compared to the ADI-15878–EBOV GP structure. CDR H3 makes extensive contacts with this glycan, and glycan contacts account for all of the interactions between GP and CDR L2 as well as framework region L3.

We determined the crystal structure of ADI-15878’s fragment antigen binding (Fab) in complex first with Ebola virus (EBOV) GP_CL_ and then again in complex with Bundibugyo virus (BDBV) GP_CL_. The structure of the ADI-15878–EBOV GP_CL_ complex was determined to 4.25-Å resolution, while the ADI-15878–BDBV GP_CL_ complex was determined to 4.75-Å resolution (Fig. 1). Each structure was solved by molecular replacement using the previously published EBOV GP_CL_ structure as a search model (Protein Data Bank [PDB] 5HJ3) (35). The EBOV GP_CL_ complex was refined to an *R*_work_/*R*_free_ of 28%/32.3%, and the BDBV GP_CL_ complex was refined to an *R*_work_/*R*_free_ of 30.6%/31.1% (see Table S1 in the supplemental material). BDBV and EBOV GPs share 64.7% sequence identity overall; however, they are 88% identical in the GP_CL_ core. Thus, it is not surprising that the two trimeric structures align with a C_α_ root mean square deviation (RMSD) of 0.59 Å over 786 residues. Primary sequence differences between EBOV and BDBV GP_CL_ concentrate in the N-terminal tail of GP2 which is not visible in these structures; however, upon comparison of surface electrostatics, an electronegative trough is observed beneath the fusion loop of BDBV GP that is largely absent in EBOV GP (see Fig. S1 in the supplemental material).

10.1128/mBio.01674-18.6 TABLE S1 Crystallographic data statistics. Download Table S1, DOCX file, 0.01 MB. Copyright © 2018 West et al. 2018 West et al. This content is distributed under the terms of the Creative Commons Attribution 4.0 International license.

10.1128/mBio.01674-18.1 FIG S1 The electrostatic surface of EBOV versus BDBV GP. (A and B) The electrostatic surface of EBOV GP (A) has a more basic cavity beneath the fusion loop compared to BDBV GP (B) due to the amino acid differences N61S, Q188E, and A189T in EBOV versus BDBV. Download FIG S1, TIF file, 7.30 MB. Copyright © 2018 West et al. 2018 West et al. This content is distributed under the terms of the Creative Commons Attribution 4.0 International license.

The ADI-15878 binding interface on both EBOV and BDBV GP is made up of approximately 65% heavy-chain contacts and 35% light-chain contacts. Roughly 37% of the overall binding interface involves complementarity-determining region (CDR) H3. Binding of ADI-15878 buries 1,490 Å^2^ of surface area on GP. The Fab binds GP in an orientation perpendicular to the viral membrane with the heavy chain proximal to the viral membrane and the light chain above (Fig. 1). Alanine scanning of the ADI-15878 antibody indicates that CDR H3 residues D95 and W99 are critical for binding and neutralization of each of EBOV, BDBV, Taï Forest ebolavirus (TAFV), Sudan virus (SUDV), and Reston virus (RESTV) GP-bearing particles (15). Interestingly, CDR H3 residue D95 is not involved in the binding interface, but instead appears to play a structural role in CDR H3, forming a hydrogen bond to the backbone nitrogen of A33 of CDR H1 (Fig. S2). CDR H3 residue W99, however, makes a direct contact to GP, packing between the base of the completely conserved GP glycan at N563 and conserved Q560 in HR1_A_ of GP2 in both EBOV and BDBV structures (Fig. S2). Heavy-chain (HC) monoclonal antibody (MAb) residues 27, 32, 100, 101, 102, 103, and 105 and light-chain (LC) residues 31, 32, 50, 51, 52, 53, 66, 67, and 71 also contact the N563 glycan. This glycan is present in both structures and is clearly visible in the higher-resolution ADI-15878–EBOV GP structure. Recognition of glycan has been noted by numerous neutralizing antibodies against the heavily glycosylated HIV-1 envelope (39–45). Here, although recognition of the N563 glycan spans several CDRs and buries ∼505 Å^2^, or ∼44% of the total buried surface area, the glycan appears instead to be accommodated by ADI-15878, rather than required by ADI-15878: the MAb exhibits improved activity against GPs from which this glycan has been mutagenically deleted (15).

10.1128/mBio.01674-18.2 FIG S2 Roles of crucial residues D95 and W99. The ADI-15878 mutations D95A and W99A each abrogate the ability of ADI-15878 to neutralize GP. Residue D95 appears to play a structural role, as it does not directly contact GP, but it does form a hydrogen bond with the amide backbone of A33. Residue W99, however, plays an integral role in the binding interface by inserting between the GP2 N-terminal tail and GP2 HR1_A_ at N563 and Q560. Download FIG S2, TIF file, 4.89 MB. Copyright © 2018 West et al. 2018 West et al. This content is distributed under the terms of the Creative Commons Attribution 4.0 International license.

**FIG 2:**
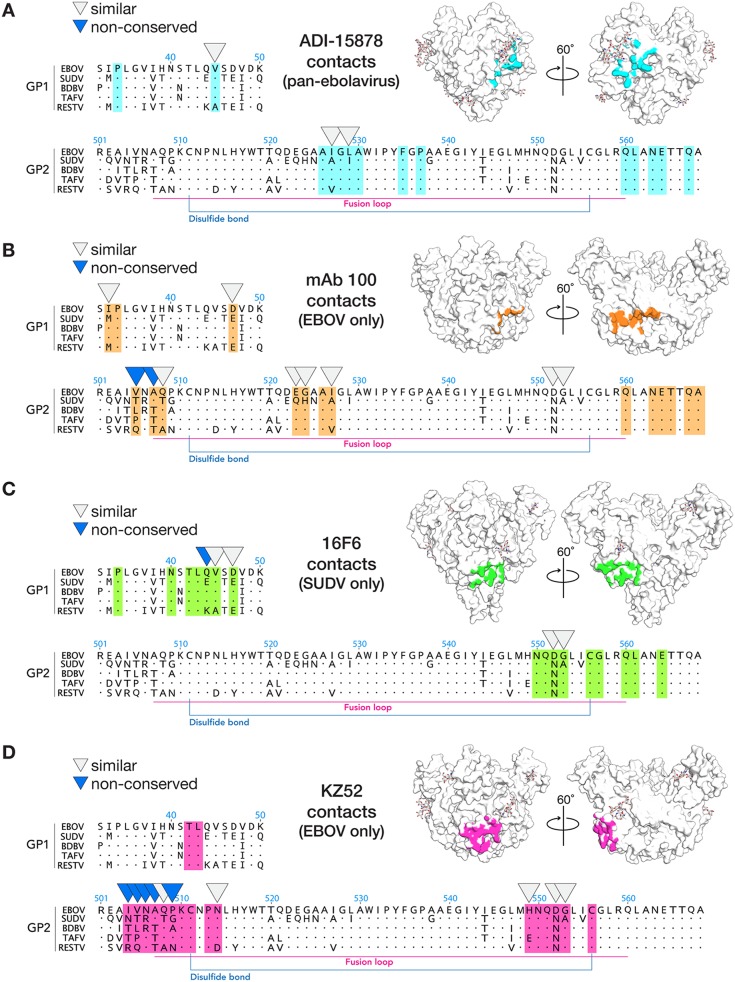
Comparison of the footprint of broadly neutralizing ADI-15878 with monospecific antibodies. In sequence alignments, footprint residues are highlighted, residues that are not identical, but similar across the ebolaviruses are indicated by light gray arrowheads, and residues that differ significantly across the ebolaviruses are indicated by blue arrowheads. (A) Footprint of ADI-15878 (light blue) on GP (white). ADI-15878 recognizes only 100% conserved or highly similar residues among the ebolaviruses. (B) The footprint of MAb100 (orange), like that of ADI-15878, is also a quaternary epitope; however, MAb100 binds to the top of the nonconserved GP2 N-terminal tail. (C) Footprint of the SUDV-specific MAb 16F6 (green) in complex with SUDV GP (white). (D) Footprint of EBOV-specific MAb KZ52 (pink), which binds the GP2 N-terminal peptide and the base of the GP complex.

ADI-15878 targets a quaternary epitope that reaches from the base region of one GP1/GP2 protomer to the fusion loop of a neighboring protomer within the GP trimer (Fig. S3). In this interaction, CDR H2 contacts the β2 strand of GP1 and the HR1_A_ helix of GP2 in the base region of GP monomer A, CDR H3 contacts the top of HR1_A_ and the glycan at N563, and CDRs L1 and L3 bind the GP2 fusion loop of the neighboring GP monomer B (Fig. S3). This quaternary mode of recognition is distinct from that of monospecific antibodies KZ52 (25), 2G4, 4G7 (37), and 16F6 (27) which also bind the base of GP but recognize only single GP monomers at a time. Instead, the footprint of ADI-15878 more closely resembles that of Ebola virus-specific human survivor MAb100 (36) which also spans a quaternary epitope, bridging the base region of one monomer to the fusion loop of a neighboring monomer (Fig. 2).

10.1128/mBio.01674-18.3 FIG S3 Overview of the ADI-15878–GP_CL_ binding interface. GP2 is shown in white, and GP1 is gray. The ADI-15878 light chain is light blue, and the heavy chain is dark blue. CDR interactions with EBOV GP_CL_ are indicated. CDRs H3, L3, L1 and framework region 3 (FRL3) interact with the conserved glycan linked to N563. Download FIG S3, TIF file, 5.67 MB. Copyright © 2018 West et al. 2018 West et al. This content is distributed under the terms of the Creative Commons Attribution 4.0 International license.

The key difference between the epitopes of monospecific MAb100 and pan-ebolavirus ADI-15878, however, is that the footprint of MAb100 includes the N-terminal tail of GP2 (Fig. 2B). MAb100 binds directly to the tail, and in the complex, the tail is tethered to the side of GP and bound into and covering the N-terminal (N-term) pocket of GP (36). In contrast, ADI-15878 displaces the N-terminal tail of GP2 and instead binds underneath and into the N-term pocket itself. Recognition of the pocket versus the tail is likely key to its broad specificity: the N-term pocket is highly conserved, while the N-term tail of GP2 is the most divergent region of GP2.

Indeed, the vast majority of the structurally characterized base-binding MAbs against EBOV also interact with the tethered GP2 N-term tail, including the monospecific KZ52 (25), c4G7, c2G4 (37), MAb100 (36), and bispecific (EBOV and BDBV) ADI-15946 (46). The only exception is the monospecific SUDV MAb, 16F6 (27, 38). In structures of MAb 16F6 bound to either SUDV Gulu or SUDV Boniface GPs, the SUDV GP2 N-terminal tail is displaced and disordered and 16F6 binds into the N-term pocket underneath it. It is unclear whether binding of MAb 16F6 displaces the tail from the SUDV GP core or whether the N-terminal tail is normally untethered/disordered in SUDV even without antibody binding. In EBOV, I504 of the tail binds into the hydrophobic pocket. SUDV, however, bears an Asn at this site that may prevent hydrophobic interactions and may disallow or discourage tethering.

Although ADI-15878 is able to react with and neutralize particles bearing the GPs of all known ebolaviruses, it is unable to bind or neutralize cuevaviruses or marburgviruses—other members of the Filovirus family (15). The ebolaviruses and cuevaviruses are ∼52% identical in the GP_CL_ core (outside the glycan cap and mucin-like domain) and 58% identical and 21% similar within the ADI-15878 footprint. Although there is not yet any structural information available for a cuevavirus GP, analysis of the primary sequence suggests that the N-terminal tail of GP2 may be considerably different in cuevaviruses versus ebolaviruses. Whereas the ebolaviruses have an eight-residue tail between the conserved C511 and the furin cleavage site at R501, cuevaviruses have ∼40 residues between these two landmarks (47). The exceptionally long N-term tail in cuevaviruses may prevent ADI-15878 from accessing the N-term pocket and thereby render it unable to bind or neutralize.

**FIG 3:**
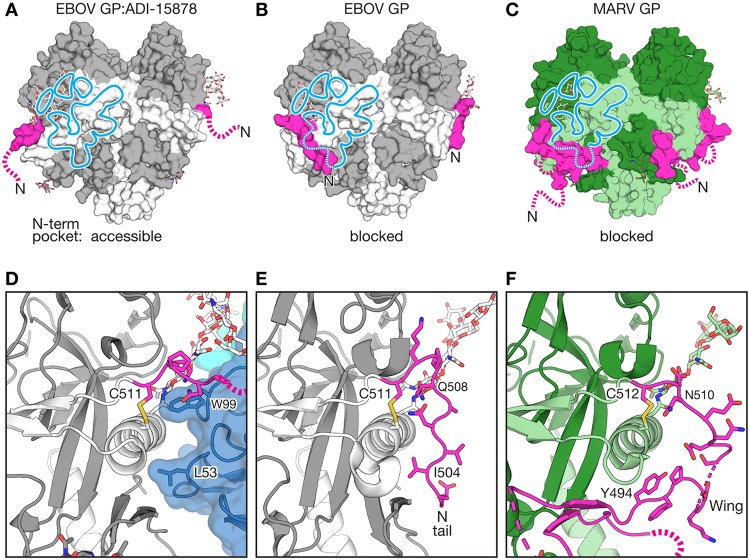
Position of the GP2 N-terminal peptide and Marburg virus-specific wing relative to the ADI-15878 footprint. (A) ADI-15878-bound EBOV GP, in which the N-terminal GP2 tail (pink) is lifted from the GP core and disordered (broken line). The footprint of antibody ADI-15878 is indicated by the light blue line. GP1 is shown in gray, and GP2 is white. (B) In EBOV GP without bound receptor or antibody (26) (pictured; PDB 5JQ3) and all other EBOV GP-antibody complexes, the N-term GP2 tail is visible (pink) and tethered along the GP core, occupying the lower portion of the ADI-15878 footprint (blue). The GP glycan cap and HR2 are not shown for simplicity. (C) In Marburg virus (MARV) GP (PDB 6BP2), a MARV-specific wing domain (also pink) hugs the GP core, also in the space that would be occupied by the ADI-15878 footprint, with GP2 N-terminal tail (pink broken line) extending outward and disordered. MARV GP1 is dark green, and GP2 is light green. (D) ADI-15878 residues L53 of CDR H2 and W99 of CDR H3 bind the GP core in a site previously occupied by the lifted GP2 N-terminal peptide (pink). The bound ADI-15878 heavy chain is dark blue and the light chain is light blue. The glycan attached to N563 is shown as sticks. (E) The standard position of the GP2 N-terminal peptide lining the outside of the GP core. GP2 I504 occupies the position of ADI-15878’s L53. (F) The extensive MARV wing domain wraps about the outside of the MARV GP core in the site that would be occupied by ADI-15878. The glycan equivalent to that of EBOV N563 (MARV N564) is illustrated in green stick representation.

The ebolaviruses and marburgviruses are 35% identical in the GP_CL_ core and 57% identical and 29% similar within the ADI-15878 footprint (Fig. S4). We observe two structural differences between the *Ebolavirus* and *Marburgvirus* (MARV) genera that may account for the inability of ADI-15878 to function as a pan-filovirus, rather than pan-ebolavirus antibody. The first site involves a Q560/R561 (EBOV/MARV) polymorphism in GP2 HR1. Q560 in both EBOV and BDBV contacts the essential residue W99 of ADI-15878’s CDR H3. The second, starker difference lies in the N-term region of GP2. In the ebolaviruses, the N-term tail of GP2 begins at the 100% conserved disulfide bond between C511 and C556 and extends eight amino acids down along the side of GP, packing into the N-term pocket formed by β2 of GP1 and HR1_A_ of GP2 (Fig. 3A and B). The marburgviruses, however, encode an additional region of GP2 called the wing domain that begins at the conserved disulfide bond and then extends 20 amino acids before it forms two beta strands to pack against the GP1 core in an equivalent position, and perhaps equivalent role, to ebolavirus GP1 β1-β2 (Fig. 3C) (48). While the ebolaviruses encode a free GP2 N terminus (the tail) that can both interact with the N-term pocket and dissociate from it to enable ADI-15878 binding, the marburgviruses do not have a free GP2 N terminus in the same place. Instead, in the marburgviruses, the marburgvirus-specific wing domain is covalently linked to the corresponding disulfide, and no free peptide can dissociate from the N-terminal pocket (Fig. 3D to F). Thus, the marburgvirus wing domain likely acts as a steric hindrance to binding by ADI-15878.

10.1128/mBio.01674-18.4 FIG S4 ADI-15878 epitope conservation. (A and B) The epitope of ADI-15878 (blue) is mapped onto the surface of the ADI-15878–EBOV GPCL (A) or the apo EBOV GP (B) (PDB 5JQ3) structures. Structures are shown in surface representation and colored according to conservation among the ebolaviruses. The apo EBOV GP structure shows the low conservation in the N-terminal tail of GP2 that buries part of the ADI-15878 epitope (dotted line). (C) The epitope of ADI-15878 mapped onto the surface of MARV GP (PDB 6BP2) shows that the epitope includes mainly conserved/similar residues and the glycan at N564. Like apo EBOV GP, MARV GP has an N-terminal tail of GP2 that obscures a portion of the epitope (dotted line). Unlike the free N terminus of EBOV GP2, however, the equivalent section in MARV GP is less likely to accommodate binding of ADI-15878, as it is covalently linked to the GP core. Download FIG S4, TIF file, 4.72 MB. Copyright © 2018 West et al. 2018 West et al. This content is distributed under the terms of the Creative Commons Attribution 4.0 International license.

**FIG 4:**
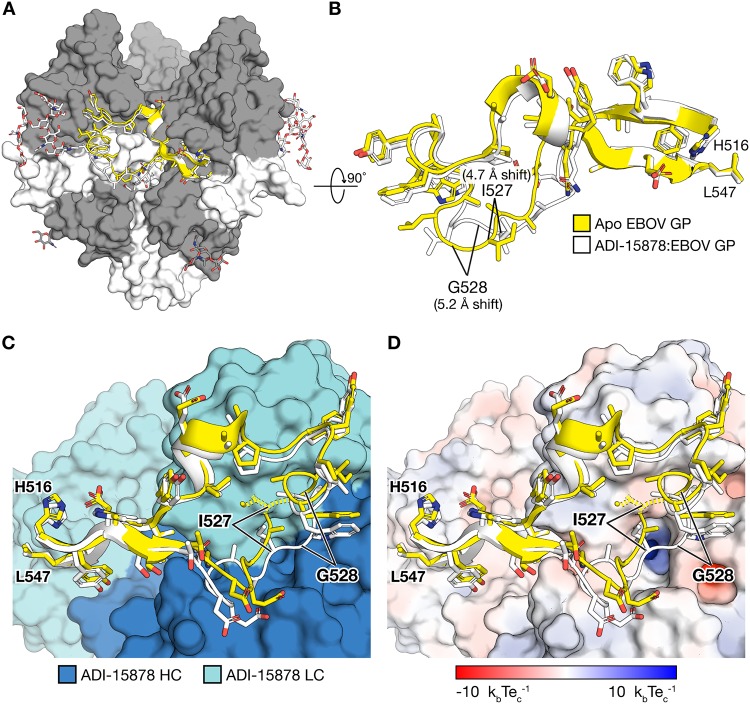
The internal fusion loop of GP2 shifts inward when bound by ADI-15878. (A) EBOV GP1 and GP2 are dark and light gray, respectively, with visible glycans illustrated in ball and stick. The fusion loop is illustrated in the standard conformation (yellow) and ADI-15878-bound conformation (white). (B) The shift in position, particularly at G528, is noted when looked at from the top of the spike down (a 90° rotation about the *x* axis) in the zoomed-in view of the internal fusion loop. (C) Interaction of ADI-15878 with the fusion loop I527 (yellow dotted line) in the standard conformation of the fusion loop sterically interferes with the bound conformation of the ADI-15878 light chain. In the ADI-15878-bound conformation (white), the fusion loop is adjusted so that I527 is accommodated between light chains (LC) (light blue) and heavy chains (HC) (dark blue). (D) Electrostatic surface of ADI-15878 with a limit of 10 k_b_Te_c_^−1^ (k_b_ is Boltzmann’s constant, T is temperature, and e_c_ is the charge of an electron) indicated in blue and red, respectively. The position of G528 is noted. Escape mutation G528E may introduce an unfavorable charge clash with the somewhat acidic outer surface of the antibody. The basic charge in the nearby pit is buried.

In the ADI-15878–GP complexes (with EBOV GP and with BDBV GP), the fusion loop adopts a conformation that differs from that observed in all Ebola virus GP structures determined thus far, whether free (26) or in complex with antibody (25, 35–37). In the ADI-15878-bound conformation, the C_α_ of GP2 residue G528 moves 5.2 Å in toward the GP core, and the C_α_ of I527 also shifts 4.7 Å from its unbound conformation (Fig. 4A and B). This site is a known escape mutant of ADI-15878 (G528E) (15). Mutation of position 528 to any residue other than Gly may prevent antibody binding by disallowing the conformational readjustments of the fusion loop required for ADI-15878 interaction. A requirement for glycine-permitted flexibility at that site may constitute a vulnerability for this antibody: modeling suggests that ADI-15878 cannot bind the fusion loop in its typically observed conformation due to steric hindrance with fusion loop residue I527 (Fig. 4C and D).

## DISCUSSION

Here we describe crystal structures of pan-ebolavirus human antibody ADI-15878 in complex with both EBOV GP and BDBV GP. This work first reveals that BDBV and EBOV GPs have similar folds and organization, with slight differences observed in the electrostatics of the cavity beneath the GP2 fusion loop. Second, this work reveals that ADI-15878 achieves its unique broad specificity and activity by binding into a conserved hydrophobic pocket at the base of the GP structures. Although multiple other monospecific antibodies have also been identified to bind the base region of Ebola virus GP, all of these antibodies interact with the N-terminal polypeptide of the GP2 subunit that covers the pocket. The N-terminal peptide is the most divergent part of GP2, while the hydrophobic pocket underneath is highly conserved. By binding underneath the peptide into the conserved pocket, ADI-15878 is able to bypass the species-specific polymorphisms that limit reactivity of other antibodies.

Interestingly, so far only one other antibody has been reported to bind into this pocket, 16F6 (27, 38). Yet 16F6 is specific for SUDV and does not bind EBOV or BDBV. Modeling suggests that the limited reactivity of 16F6 may be due to SUDV-specific differences in the flexibility of the N-terminal tail, particularly at positions 504 and 509. At position 504, EBOV encodes an Ile which binds into the hydrophobic pocket, while SUDV encodes instead a polar Asn. At position 509, EBOV encodes a Pro which limits conformational mobility of the N-terminal peptide. SUDV instead encodes a Gly which enhances conformational mobility of the peptide. The Pro-containing N-terminal tail of EBOV may be unable to lift high enough or frequently enough to accommodate 16F6 binding. The specificity of 16F6 may also be the result of other epitope residue differences such as D552N or Q44E in EBOV versus SUDV. It is also possible that there are more global structural differences in the recognition of the epitopes due to differences in the scaffolding of the paratopes in ADI-15878 versus 16F6.

The N-terminal tail of GP2 may play a role in regulation of membrane fusion. We note that for EBOV, it is tacked down onto the GP core in all structures except in this complex with ADI-15878 and a prior complex with its receptor NPC1 (22). Conformational changes transmitted in GP as a result of NPC1 binding may lift the N-terminal tail and encourage the dissociation of the GP2 fusion loop from the GP1 core. KZ52 and other base-binding antibodies lock down the GP2 N terminus and prevent it from untethering. In contrast, ADI-15878 uses a strategy to bind underneath the GP2 N-terminal tail and replace the hydrophobic tail-pocket interactions with its own hydrophobic antibody-pocket interactions, potentially inhibiting any as yet undiscovered downstream steps in the GP fusion pathway.

ADI-15878 likely neutralizes by anchoring the HR1 of GP2 to the GP core through contacts between HR1 and CDR H3 and hydrophobic packing of CDR H2 into the β1-β2 N-term pocket. Additionally, contacts between CDRs H3/L3 and the paddle of the fusion loop may prevent the fusion loop from unravelling from the GP core to seek the target cell membrane.

**FIG 5:**
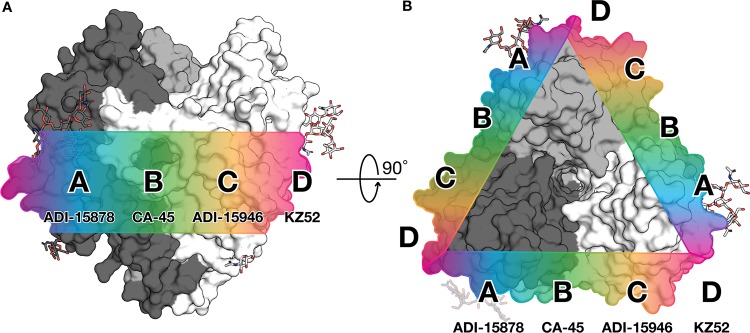
Continuum of antibody epitopes at the GP “waist.” Sites A to D are shown. (A) The approximate locations of the various epitopes are shown mapped along the surface of GP in a side view. (B) A top-down view of the GP trimer highlights the repeating nature of these epitopes around the circumference of GP.

Antibodies against Ebola virus have been previously categorized into “base-binding” and fusion loop-binding epitope groups (14, 17, 25, 49, 50). The ADI-15878–GP_CL_ complex and other new structures now make it clear that there is a continuum of antibody epitopes from base binding to fusion loop sites and everywhere in between. Within this spectrum along the “waist” of GP are ADI-15878-like footprints (site A, epitopes that overlap with those of ADI-15878, MAb100, and perhaps KZ52), CA-45-like footprints (17) (site B, epitopes that overlap with those of ADI-15878 and ADI-15946, but not KZ52), ADI-15946-like footprints (46) (site C, epitopes that overlap with those of ADI-15946 and perhaps KZ52), and KZ52-like footprints (25) (site D, epitopes that overlap with those of ADI-15878, ADI-15946, and KZ52) (Fig. 5). Currently characterized antibodies that interact with the GP2 N-terminal tail at site A or D are monospecific, whereas those that instead avoid the N-terminal tail at sites A-C are more broadly reactive. Future classification of base- and fusion loop-binding antibodies into waist-binding groups using a competition assay based on these regions may help in the selection of antibodies for immunotherapeutic cocktails. For example, structural evidence suggests that ADI-15878 and ADI-15946 may not compete with each other even though they have both been characterized to compete with KZ52 (see Fig. S5 in the supplemental material) (15).

10.1128/mBio.01674-18.5 FIG S5 Steric competition of antibodies that target the spectrum of binding sites including and between the base and fusion loop. Such information may be important in characterization of future antibodies and in cocktail selection. The epitope of ADI-15878 (C) (blue) does not overlap with that of ADI-15946 (A) (orange), even though both MAbs compete with KZ52 (B). An alignment of all three to the same GP (D) shows the spectrum of the base epitope as it fades into the fusion loop epitope on either side of KZ52. The trimeric nature of GP presents these epitopes in a repeating pattern around the three sides of GP (E). (F) Repeat of Fig. 5, illustrating locations of these antibodies in the GP waist: ADI-15878 in site A (blue), ADI-15946 in site C (orange,) and KZ52 in site D (pink). Download FIG S5, TIF file, 17.67 MB. Copyright © 2018 West et al. 2018 West et al. This content is distributed under the terms of the Creative Commons Attribution 4.0 International license.

Binding of the conserved pocket underneath the GP2 tail (site A; ADI-15878) may be a more broadly applicable strategy for identification of a pan-filovirus MAb that would also cross-react to Marburg virus. An antibody against an ADI-15878-like epitope but shifted slightly away from the Marburg virus-specific wing domain may be able to achieve this broader specificity. Further, deletion of the GP2 N-terminal peptide from immunogens may assist elicitation of more broadly reactive antibodies and may be a useful strategy in designing broadly protective vaccines effective against any of the ebolaviruses with outbreak potential.

## MATERIALS AND METHODS

### Protein expression and purification.

Expression and purification of EBOV and BDBV GP_CL_ was performed as described previously (35). Briefly, Ebola virus and Bundibugyo virus GPs (lacking the mucin domain residues 312 to 462) were produced by stable expression in Drosophila melanogaster S2 cells. Effectene (Qiagen) was used to transfect S2 cells with a modified pMT-puro vector plasmid containing the GP gene of interest, followed by stable selection of transfected cells with 6 µg/ml puromycin. Cells were cultured at 27°C in complete Schneider’s medium for selection and then adapted to Insect Xpress medium (Lonza) for large-scale expression in 2-liter Erlenmeyer flasks. Secreted GP ectodomain expression was induced with 0.5 mM CuSO_4_, and supernatant was harvested after 4 days. EBOV and BDBV GPs were engineered with a double Strep-tag at the C terminus to facilitate purification using Strep-Tactin resin (catalog no. 2-1201-010; Qiagen) and then further purified by Superdex 200 (GE) size exclusion chromatography (SEC) in 10 mM Tris-buffered saline (TBS) (Tris-HCl [pH 7.5], 150 mM NaCl). EBOV and BDBV GP_CL_ were produced by incubation of 1 mg each GP with 0.02 mg thermolysin overnight at room temperature in TBS containing 1 mM CaCl_2_ and purified using Superdex 200 SEC. Thermolysin cleavage does not remove the GP2 N-terminal tail. This tail was visible in thermolysin-cleaved EBOV GP_CL_ in complex with KZ52 and ADI-15946 (35, 46).

ADI-15878 Fab used for crystallization experiments was cloned into a modified pMT-puro vector with a heavy-chain C-terminal Strep-tag, and then expressed and purified according to the protocol for GP_CL_ with the exception that SEC was performed with a Superdex 75 column (GE) (35).

### Crystallography and structure determination.

Trimeric EBOV and BDBV GP_CL_ were complexed with ADI-15878 Fab fragments, and the resulting complex was then purified via SEC. The purified EBOV GP_CL_–ADI-15878 Fab and BDBV GP_CL_–ADI-15878 Fab complexes were concentrated to an *A*_280_ of 6.4 in TBS. The crystal drops consisted of a 1:1 ratio of protein-well solution. ADI-15878–EBOV GP crystals grew over the course of 3 weeks in 100 mM MgCl_2_, 100 mM HEPES pH 7.5, and 10% PEG 4000. ADI-15878–BDBV GP crystals grew over the course of 2 weeks in 2% Tacsimate (pH 8.0), 100 mM Tris (pH 8.5), and 16% polyethylene glycol 3350 (PEG 3350). Crystals from both conditions were cryoprotected with 15% ethylene glycol and flash frozen in liquid nitrogen for storage and shipping. Diffraction data were collected remotely on Advanced Photon Source (APS) beamline 23ID-B on a pilatus 6M detector (51–54). Data were processed using XDS (55, 56), and the structure was determined using molecular replacement with PHASER (57), within the CCP4 suite (58), using the structure of EBOV GP_CL_ (PDB 5HJ3) as an initial search model (35). Iterative rounds of model building were performed using Coot (35), and each round was refined with Phenix (59). Five percent of the data were set aside prior to refinement for the *R*_free_ calculations for each data set (60). Side chains were built into observed density wherever possible. When the map lacked density for complete side chains, side chains were modeled into any observable density according to their most common rotamer while taking into account high-resolution structures of EBOV GP. The statistics and stereochemistry of the crystal structure were checked using the MolProbity server (60, 61). Structural figures were rendered using Open Source PyMOL (PyMOL Molecular Graphics System, version 1.7.0.0; Schrödinger, LLC).

### Alignment and visualization of filovirus sequences.

Alignment was performed using clustalomega on uniprot (62, 63) with the following virus protein sequences: *Zaire ebolavirus*, Q05320; *Bundibugyo ebolavirus*, B8XCN0; *Sudan ebolavirus*, Q66814; *Taï Forest ebolavirus*, Q66810; *Reston ebolavirus*, Q66799; *Lake Victoria marburgvirus*, Q1PDC7. Sequence conservation was numbered according to EBOV GP and visualized using the Espript server (http://espript.ibcp.fr) and colored according to the percent equivalent scoring function with a cutoff of 70% (64).

### Accession number(s).

Atomic coordinates and structure factors have been deposited into the Protein Data Bank under accession numbers 6EA7 for the EBOV GP_CL_–ADI-15878 complex and 6EA5 for the BDBV GP_CL_–ADI-15878 complex.
