# Supplementary material for: Structural Basis of Pan-Ebolavirus Neutralization by a Human Antibody against a Conserved, yet Cryptic Epitope
Source: mBio. 2018 Sep 11;9(5):e01674-18. doi: 10.1128/mBio.01674-18 (PMC6134094; doi:10.1128/mBio.01674-18)
Supplement: TABLE S1 [file mbo004184064st1.docx]

**Supplemental Table 1.** Crystallographic data statistics.

|  | **EBOV GPCL – ADI-15878**  PDB ID 6EA7 | **BDBV GPCL – ADI-15878**  PBD ID 6EA5 |
| --- | --- | --- |
| Number of Datasets Merged | 10 | 1 |
| Resolution^a^ (Å) | 56.08– 4.25 (4.40 – 4.25) | 43.77- 4.75 (5.31-4.75) |
| Space group | P3_1_21 | P3_1_21 |
| Unit cell (Å)  (°) | 152.78 152.78 247.69  90 90 120 | 151.63 151.63 247.10  90 90 120 |
| Total Reflections^a^  Unique Reflections^a^  Reflections used in Refinement^a^ | 4,193,311 (702,866)  24,218 (4,317)  24,151 (2,361) | 111,058 (31,082)  17,080 (4,757)  17,006 (1,657) |
| Multiplicity^a^ | 173.1 (162.8) | 6.5 (6.5) |
| Completeness^a^ (%) | 99.9 (100.0) | 99.7 (100.0) |
| I/σ(I) ^a^ | 10.6 (2.1) | 10.1 (1.1) |
| Rmerge^a^ | 109 (1,655) | 0.09 (1.90) |
| Rpim^a^ | 8.06 (125) | 0.06 (1.21) |
| CC_1/2_ ^a^ | 1.00 (0.56) | 1.00 (0.53) |
| Wilson B (Å^2^) | 133 | 273 |
| R_work_ (%)  R_free_ (%) | 28.0  32.3 | 29.5  30.5 |
| Reflections used in Refinement^a^ | 24,151 (2,361) | 17,006 (1,657) |
| RMSD (bonds) (Å) | 0.00 | 0.01 |
| RMSD (angles) (°) | 0.68 | 1.14 |
| Ramachandran favored (%) Ramachandran outliers (%) | 97.4  0 | 96.7  0 |
| Average B-factor (Å^2^) | 173 | 204 |

a Values in parentheses are for the highest-resolution shell
