# Supplementary figures and images for: Structural Basis of Pan-Ebolavirus Neutralization by a Human Antibody against a Conserved, yet Cryptic Epitope
Source: mBio. 2018 Sep 11;9(5):e01674-18. doi: 10.1128/mBio.01674-18 (PMC6134094; doi:10.1128/mBio.01674-18)

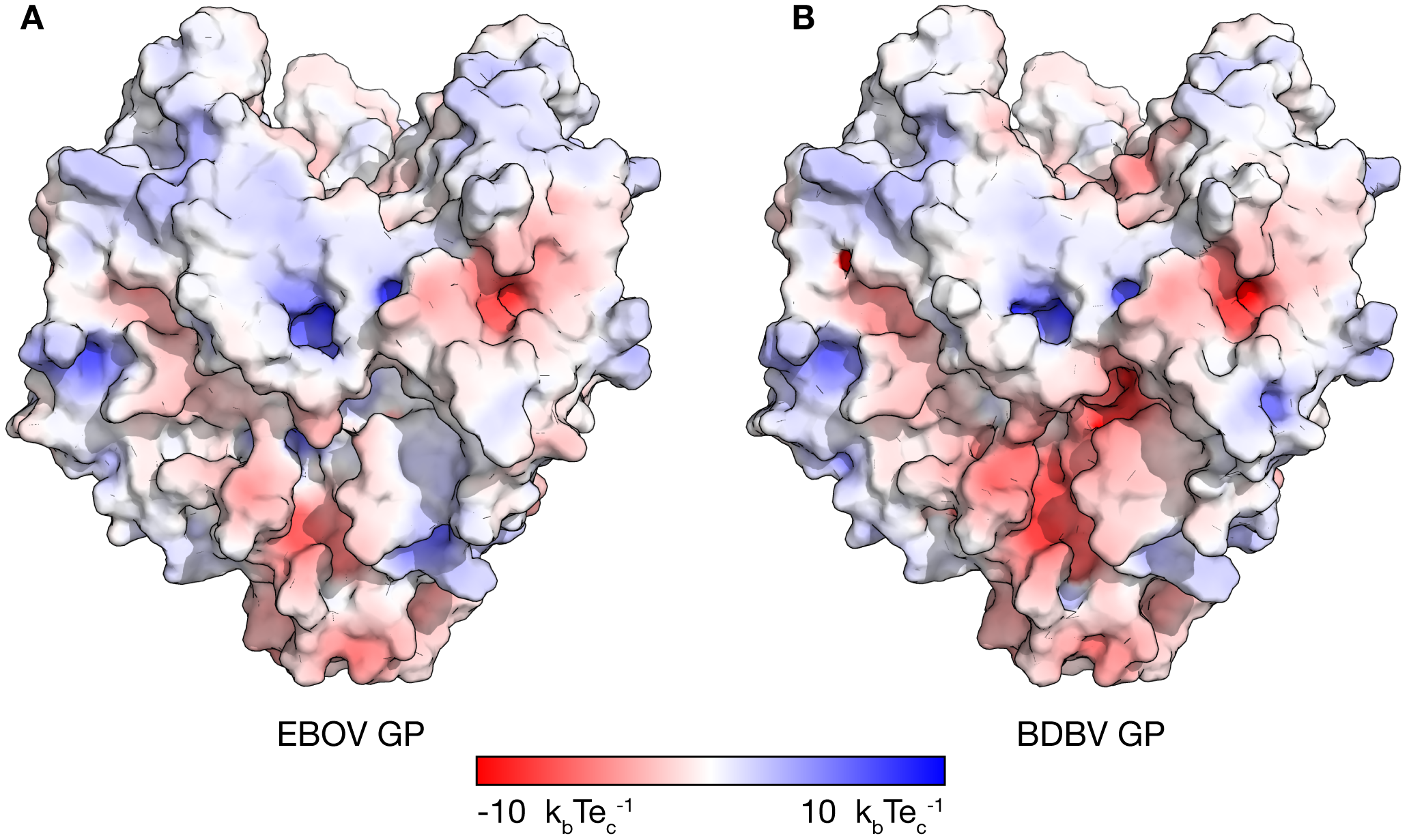

Supplement: FIG S1 [file mbo004184064sf1.tif]

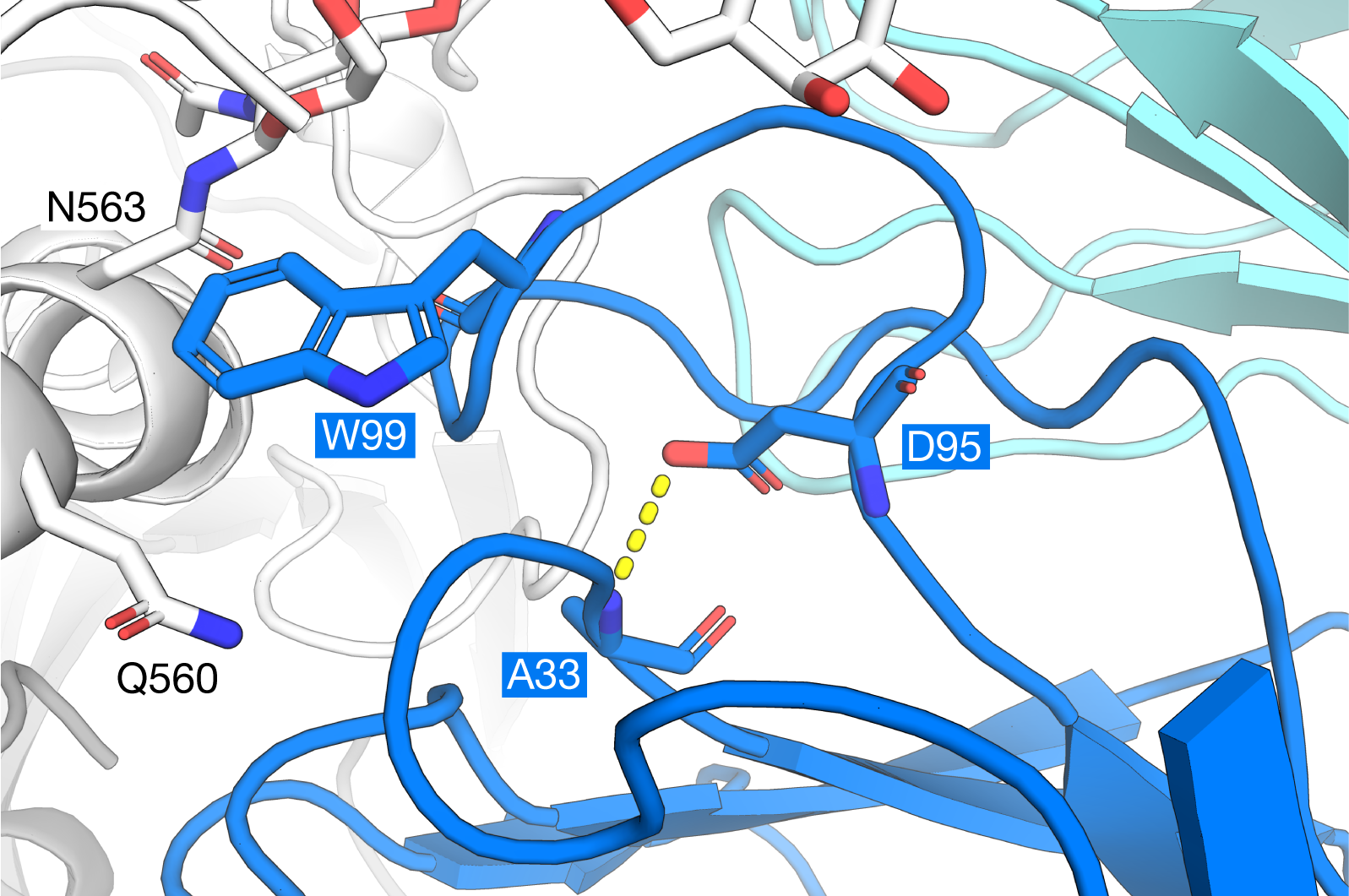

Supplement: FIG S2 [file mbo004184064sf2.tif]

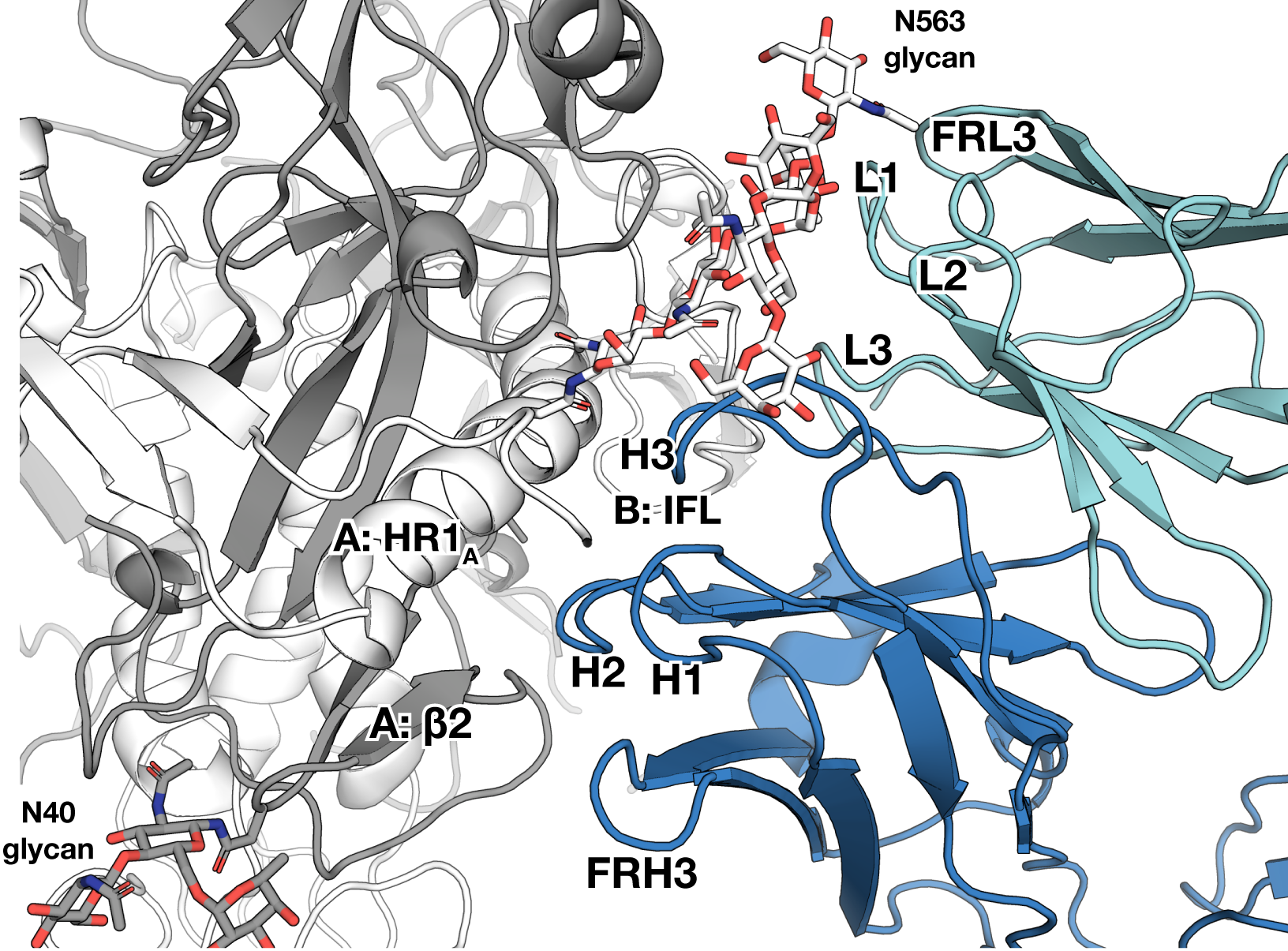

Supplement: FIG S3 [file mbo004184064sf3.tif]

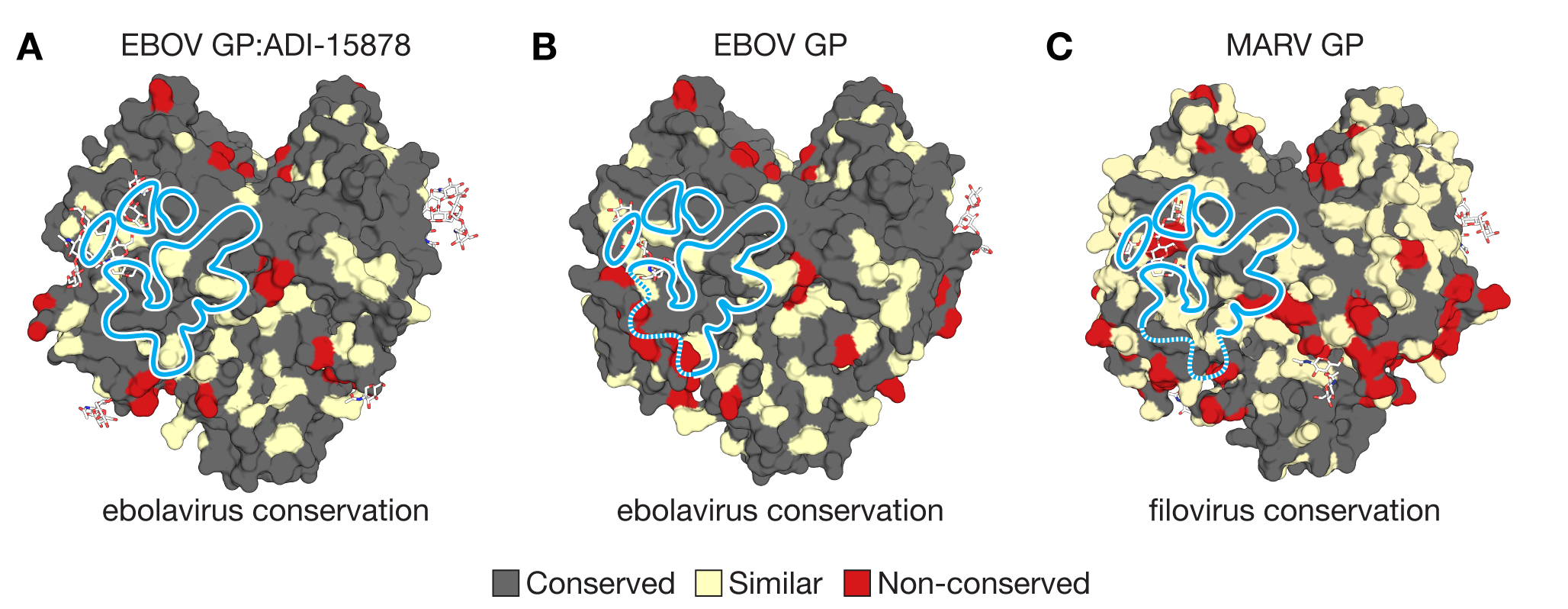

Supplement: FIG S4 [file mbo004184064sf4.tif]

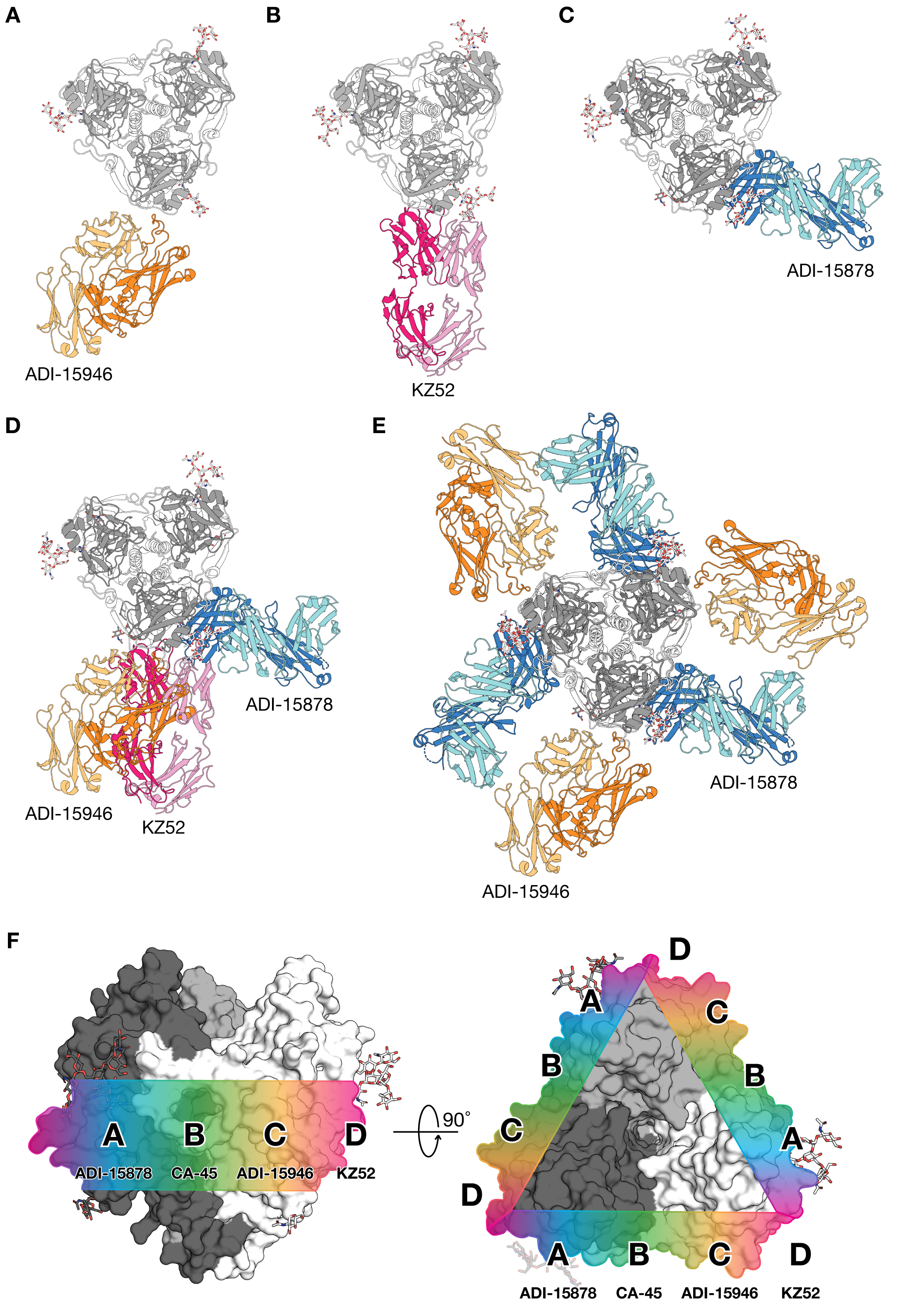

Supplement: FIG S5 [file mbo004184064sf5.tif]
